# Medication-related factors associated with health-related quality of life in patients older than 65 years with polypharmacy

**DOI:** 10.1371/journal.pone.0171320

**Published:** 2017-02-06

**Authors:** Alonso Montiel-Luque, Antonio Jesús Núñez-Montenegro, Esther Martín-Aurioles, Jose Carlos Canca-Sánchez, Maria Carmen Toro-Toro, José Antonio González-Correa, on behalf of the Polipresact Research Group

**Affiliations:** 1 San Miguel Health Center, Costa del Sol Primary Healthcare District, Andalusian Health Service, Málaga, Spain; 2 North Málaga Health Area, Andalusian Health Service, Málaga, Spain; 3 La Roca Health Center, Málaga-Guadalhorce Primary Healthcare District, Andalusian Health Service, Málaga, Spain; 4 Costa del Sol Health Agency, Andalusian Health Service, Marbella (Málaga), Spain; 5 Campillo Health Center, North Málaga Health Area, Andalusian Health Service, Málaga, Spain; 6 Department of Pharmacology, Biomedical Research Institute of Málaga. University of Málaga, Málaga, Spain; University of Glasgow, UNITED KINGDOM

## Abstract

In the current public health framework, the importance of medication as a determinant of citizens’ health has emerged as a factor warranting special attention. Most studies investigating the relationship between medication and quality of life do so from the perspective of adherence. However, other medication-related factors identified at home visits may be associated with health-related quality of life.

**Methods and design:**

Objective: To describe the relationship between medication-related factors and the health-related quality of life in patients older than 65 years who use multiple medications (polypharmacy).

Design: Cross-sectional descriptive study.

Setting: Primary care.

Participants: Patients older than 65 years who use multiple medications (n = 375).

Measurements: The main outcome measure was health-related quality of life according to the EuroQol-5D instrument. Sociodemographic, clinical and medication-related variables were recorded during home interviews.

**Results:**

Mean age was 74.72 ± 5.59 years, and 65.5% of our participants were women. The global level of health-related quality of life according to the EQ-5D visual analog scale was 59.25 ± 20.92. Of the five EuroQol dimensions, anxiety/depression and pain were the most frequently reported, while mobility and self-care were the dimensions with the greatest impact on self-reported quality of life. Multivariate analysis indicated that functional independence was the factor most strongly associated (β = 14.27 p < 0.001) with better health-related quality of life, while illiteracy (β = −13.58 p < 0.001), depression (β = −10.13 p < 0.001), social risk (β = −7.23 p = 0.004) and using more than 10 medicines (β = −4.85 p = 0.009) were strongly associated with a poorer health-related quality of life.

**Conclusions:**

Factors inherent within the patient such as functional incapacity, cognitive impairment and social and emotional problems were the main constraints to quality of life in our study population. The number of medicines taken was negatively related with quality of life.

## Introduction

In recent decades the population of Spain has aged so much that according to United Nations estimates that by 2050 Spain will have the world’s oldest population [1, 2]. Sociodemographic changes and scientific advances in the last decades have turned terminal diseases into chronic diseases and resulted in a significant increase in life expectancy. As a result of these changes a new health care scenario has emerged, and the longevity of the elderly has increased albeit at the expense of their quality of life [3]. In this connection, within the framework of the Horizon 2020 Programme the European Union has identified “Healthy Living and Active Ageing” as a priority, in order to improve the quality of life of the elderly and help them contribute to society as they grow older [4].

Schumaker defines health-related quality of life (HRQoL) as "the subjective perception influenced by the current health status of the ability to perform activities important for the person" [5,6]. Efforts to assess HRQoL are becoming increasingly important in response to needs to examine the health status of the population and analyze the efficacy and effectiveness of health care interventions [7]. Consequently, a range of questionnaires and instruments has been developed to measure and characterize HRQoL [8, 9]. Assessments of HRQoL make it possible to evaluate the influence of health status and other associated factors on the general well-being of individuals [10]. However, studies published thus far regarding factors associated with HRQoL in people older than 65 years have reported discrepant and controversial results. Some authors have found poorer HRQoL associated with age, female sex, functional impairment, depression, chronic diseases or polypharmacy [11–17].

In the current public health framework, the importance of medication as a determinant of citizens’ health has emerged as a factor warranting special attention [18]. Most studies that have investigated the relationship between medication and quality of life have done so from the perspective of adherence [19–22]. However, other medication-related factors identified at home visits may be associated with HRQoL, but these factors have not been extensively studied to date. A better knowledge of these factors may be useful to tailor interventions to the needs of individuals in efforts to improve both care and HRQoL [23].

Accordingly, this study was designed to describe the relationship between medication-related factors and HRQoL in patients older than 65 years who use multiple medications.

## Methods

This cross-sectional descriptive study was done over a period of 1 year; the participants were patients older than 65 years with polypharmacy who were being treated at different primary care centers in the Costa del Sol Health District and North Málaga Health Area (southern Spain), which included 19 clinical management units.

### Participants

Patients were selected by stratified randomized sampling from the lists of patients older than 65 years who were using multiple medications. The lists of these patients treated at each of the 19 clinical management units were provided by the pharmacy service of each health district.

A total of 375 patients were included in the study. The sample size according to one of the independent variables related to medication use, i.e. adherence, was estimated as 344 patients (375 including enough patients to compensate for dropouts) assuming a mean standard difference in quality of life of 0.35 points between patients with low and high adherence to medication, for a 95% confidence level, with 80% power and a group ratio of 1:1.

To achieve the necessary sample size (N = 375) we selected 1125 patients older than 65 years who used multiple medications. Of these, 430 did not meet the criteria for inclusion or exclusion, 123 could not be located and 197 (17.5%) refused to participate in the study.

### Inclusion criteria

- Age ≥ 65 years.
- Polypharmacy: use of five or more medications for a period ≥ 6 months [24].
- Inclusion in the electronic prescription program (75.9% of patients during the study period) [25].

### Exclusion criteria

- Functional and/or cognitive impairment preventing autonomous management of medication (Barthel ≤ 60 or more than four errors in the Pfeiffer test).
- Inpatients at public/private institutions.
- Patients with psychiatric disorders.
- Refusal to provide written consent.
- Patients with language impairment preventing fluent communication.

### Study variables

#### Dependent variable

Health-related quality of life.

#### Predictor variables

Sociodemographic, clinical and medication-related variables.

Sociodemographic: Age, sex, place of residence, cohabitation, socioeconomic status (according to a minimum wage in Spain of 641.40 €/month): Low: <641.40 €/month; Middle: 641.40 €/month to 962.10 €/month; High: >962.10 €/month, educational level, and social risk based on the Gijón scale (S1 File). This instrument assesses social and family risk, and consists of 5 items: family situation, economic situation, housing, social relations and social support network. The cutoff score for the detection of social risk is 16.

Clinical Data: Functional assessment (Barthel index) (S1 File), cognitive assessment (Pfeiffer test) (S1 File), assessment of emotional status: anxiety/depression (Goldberg scale) (S1 File), medical conditions and history of emergency room visits.

The Pfeiffer test or Short Portable Mental Status Questionnaire (SPMSQ) provides a measurement of cognitive functioning of elderly people. The ten items in the SPMSQ cover orientation in time and place, remote memory, and general knowledge. The SPMSQ establish four categories of severity of chronic brain syndrome according to number of errors made: 0–2 errors: normal; 3–4 errors: mild cognitive impairment; 5–7 errors: moderate; 8 or more errors: severe.

The Goldberg scale (GADS) score is based on responses of ‘Yes’ or ‘No’ to nine depression and nine anxiety items, asking how respondents have been feeling in the past month. Goldberg et al. considered patients with anxiety scores of 5 or more or with depression scores of 2 or more as having a 50% chance of a clinically important disturbance.

Medication: Number of medicines, medication errors (drug-related problems [DRP] according to PCNE classification V6.2, ability to identify medicines with disease (patient’s ability to relate the medicines with the disease for which they were prescribed), presence of different brands of medicines, and medication adherence (Morisky-Green test). The Morisky Medication Adherence Scale (MMAS) is a generic self-reported medication-taking behavior scale. Although it was originally developed for the assessment of compliance in hypertensive patients, subsequently has been validated for other chronic diseases. It consists of four items with a scoring scheme of “Yes” or “No”. Adherence is considered to be present if the patient responds correctly to all 4 items.

### Measurement instrument

To measure the dependent variable HRQoL, we used the EuroQol-5D instrument, a generic HRQoL questionnaire that has been translated into Spanish and validated [26]. The EQ-5D has many applications in primary care. One of its advantages is that it can be delivered and completed rapidly and easily (2–3 min). In addition, the data it yields can be used for different purposes from the description of the overall health status by dimensions to the economic evaluation of health care services. The properties of this questionnaire have been validated both for the general population [27] and for disease groups [28], and there is an index of preference values for health states for the Spanish population [29].

The model used in this study consisted of three parts:

EQ-Index: Description of health status according to five dimensions (mobility, self-care, usual activities, pain/discomfort and anxiety/depression). Respondents are asked to choose among three levels of severity (“no problems”, “some/moderate problems” or “severe problems”) to describe their own health status”today”. For ease of presentation in the tables, the severity levels are reported as either “with problem” or “without problem”.

EQ-VAS: This visual analogue scale (VAS) is anchored at 100 at the top (best imaginable health status) and 0 at the bottom (worst imaginable health status). Respondents are asked to draw a line from 0 to whichever point of the scale best describes their health state “today”.

In the third part of the questionnaire respondents were asked to rate their current health state during the previous 12 months as better, the same, or worse.

### Data collection

One study leader per health district, area and participating center was appointed for data collection. Once stratified randomized sampling was completed, recruitment was done by the study leaders at each center or district.

In both health districts the data were collected by the same person, a fellow contract nurse with knowledge of pharmacology, trained in the methodology used in the study. Face-to-face interviews were done at the patient's home using a specifically-designed questionnaire that included all tests and variables to be assessed in the study.

### Statistical analysis

The data were analyzed with SPSS software (IBM SPSS Statistics for Windows version 22.0, Armonk, NY: IBM Corp., under license to the Central Computer System of the University of Malaga, Spain). An initial descriptive analysis of variables was performed to obtain measures of central tendency and dispersion for quantitative variables, and frequencies and percentages for qualitative variables. Student’s t test was used to identify differences between EuroQol index and EQ-VAS values with respect to the different variables, if the values met the criteria for quantitative variables regarding normal distribution and homogeneity of variance. Otherwise, the equivalent nonparametric Mann–Whitney U test was used. The chi-squared test was used for qualitative variables. One-way analysis of variance was followed by Bonferroni correction. Multivariate analysis was done by multiple linear regression in order to identify factors associated with HRQoL for both the EQ-VAS and the EQ index.

### Ethical aspects

This study was approved by the Ethics and Research Committees of the Costa del Sol Health District and North Málaga Health Area, which verified that the study was performed in accordance with all ethical standards and the Declaration of Helsinki.

Confidentiality and voluntary participation in the study were assured, and written informed consent was obtained. All the data collected were anonymous, in accordance with the provisions of Organic Law 15/1999 of 13 December on the Protection of Personal Data and the Spanish 41/2002 Act of 14 November, which regulates patients’ autonomy, rights and responsibilities in the area of clinical information and documentation.

## Results

During the 1-year data collection period a total of 375 patients who met the eligibility criteria were interviewed. Their mean age was 74.72±5.59 years, 63.5% of the sample were women and lived in the Costa del Sol District 77.9% of the cases.

**Table 1** shows the main study variables along with sample characteristics. The data for sociodemographic variables showed that 22.8% of patients lived alone, and 90.1% of patients had a middle socioeconomic level. Regarding educational level, 57.9% could read and write, 19.5% were illiterate and 16.3% had an intermediate social risk.

**Table 1:** Study variables and description of the sample.

Variables (N = 375)	Categories	n	%
**Sociodemographic variables**	** **	** **	** **
Age (years)	65–69	86	22.9
	70–74	99	26.4
	75–79	109	29.1
	≥80	81	21.6
Sex	Male	137	36.5
	Female	238	63.5
Residence	Costa del Sol	292	77.9
	North Malaga	83	22.1
Cohabitation	Alone	85	22.8
	Accompanied	290	77.2
Socioeconomic level	Low	19	5.1
	Middle	338	90.1
	High	18	4.8
Social assessment (Gijón scale)^1^	Low or normal	314	83.7
	Intermediate	61	16.3
**Clinical variables**			
BADL (Barthel test)^2^	Independent	257	68.5
	Mild dependence	50	13.3
	Moderate dependence	68	18.2
Mental assessment (Pfeiffer test)^3^	Normal	366	97.6
	Mild impairment	9	2.4
Emotional state (Goldberg scale)^4^	Anxiety	227	60.5
	Depression	128	34.1
Main pathologies	Hypertension	330	88
	Dyslipidemia	244	65.1
	Diabetes	197	52.5
ER^5^ visits (previous 12 months)	No	172	45.9
	Yes	203	54.1
**Medication variables**			
Number of medicines	≥10	196	52.3
	>10	179	47.7
Medication adherence (Morisky-Green)	Adherence	194	51.7
	Nonadherence	181	48.3
Drug-related problems (P1, P2)^6^	No	63	16.8
	Yes	312	83.2
C7.1- Patient forgot to use/take drug	No	216	57.6
	Yes	159	42.4
C.5.5- Wrong drug taken	No	363	96.8
	Yes	12	3.2
C5.6- Drug abused (unregulated overuse)	No	187	49.9
	Yes	188	50.1
C5.1- Inappropriate timing of administration and/or dosing intervals	No	142	37.9
	Yes	233	62.1
Different brands of medicines	No	59	15.7
	Yes	316	84.3
Identifies medicines with disease	No	76	20.3
	Yes	299	79.7
**Quality of life**			
Health perception today (about 1 year ago)	Better	63	16.8
	Equal	142	37.9
	Worse	170	45.3
EuroQol Visual Analogue Scale (EQ-VAS)	<60	201	53.6
	≥60	174	46.4

^1^Gijón scale (Social risk): < 10: normal or low; 10–16: intermediate; >16: high.

^2^BADL: Basic activities of daily living. Barthel scale: independent (100); mild dependence (91–99); moderate dependence (61–90).

^3^Pfeiffer: normal (0–2), mild impairment (3–4).

^4^Goldberg: anxiety subscale (≥4 risk of anxiety); depression subscale (≥6 risk of depression).

^5^ER: Emergency room.

^6^Drug-related problems (DRP) according to PCNE classification V6.2. P1: Treatment effectiveness (There is a (potential) problem with the (lack of) effect of the pharmacotherapy); P2: Adverse reactions (Patient suffers, or will possibly suffer, from an adverse drug event).

In the functional and cognitive assessment according to the Barthel scale for usual activities, average score was 96.68 ± 6.01, and 68.5% of the patients were considered independent. In terms of cognitive ability, only 9 patients (2.4%) had mild cognitive impairment according to the Pfeiffer test.

The most prevalent conditions were hypertension (88%), dyslipidemia (65.1%) and diabetes mellitus (52.5%). According to the Goldberg scale, 60.5% and 34.1% of patients were at risk of suffering from anxiety or depression, respectively. Slightly more than half (54.1%) of the respondents had been seen in the emergency room in the previous year.

A total of 47.7% of patients took more than 10 drugs daily. According to the Morisky-Green test, 48.3% of patients showed poor adherence to treatment. No differences were identified according to age, p = 0.426 or sex, p = 0.376, respectively.

We found that 83.2% of patients had some causes of DRP. Categories C5.1 (62.1%) and C5.6 (50.1%) were the most frequent causes. Different brands of the same medication were found in 84.3% of cases.

**Table 2** shows global perception of quality of life as assessed by the EQ-VAS (0–100) and EQ-Index (0–1). Men had a higher mean score than women, and patients older than 75 years obtained lower EuroQol Index values compared to the remaining patients.

**Table 2:** Mean EuroQol-5D results by age and sex.

Variable	Total	Male	Female	≤75	>75
EQ-VAS(0–100)^1^	59.25±20.92	64.19±19.10*	56.43±21.43	59.07±21.34	59.47±20.46
EQ-Index(0–1)^2^	0.65±0.19	0.72±0.17*	0.62±0.19	0.67±0.20	0.63±0.17*

^1^EQ-VAS: EuroQol Visual Analogue Scale

^2^EQ-Index: EuroQol Index.

* p < 0.05.

Among the EuroQol-5D dimensions **(Table 3)**, anxiety/depression were the most prevalent disorders (69.1%), followed by pain/discomfort (58.4%) and mobility problems (54.9%). With respect to sex, significant differences were found in all dimensions except self-care, with a higher prevalence of self-care problems in women. With regard to age, statistically significant differences were found in patients older than 75 years in the dimensions mobility, self-care and autonomy for performing usual activities.

**Table 3:** Percentage of participants with problems in each dimensions of the EQ-Index by age and sex.

Dimension	Total	Male	Female	≤75	>75
Mobility	54.9	46.7	59.7*	45.6	66.3 *
Self-care	34.4	29.2	37.4	27.7	42.6 *
Usual activities	47.2	35	54.2 *	37.9	58.6 *
Pain/discomfort	58.4	42.3	67.6 *	55.8	61.5
Anxiety/Depression	69.1	54.7	77.3 *	71.4	66.3

* p < 0.05.

**Fig 1:**
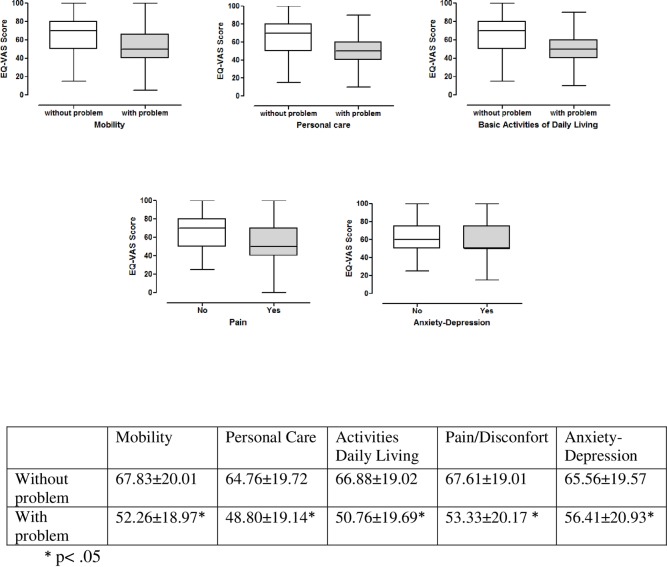
Perceived quality of life (EQ-VAS) according to different dimensions.

Regarding the influence of each EuroQol-5D dimension on self-reported quality of life assessed with the EQ-VAS (Fig 1), the presence of problems in any of the dimensions has a statistically significant impact on HRQoL compared to patients without any problems. Patients with the highest overall HRQoL score were those with no mobility problems (EQ-VAS 67.83 ± 20.05), whereas patients with the lowest score were those who had self-care problems (EQ-VAS 48.80 ± 19.14)**(Fig 1).**

**Table 4** shows the values for the EQ-VAS and EQ-Index according to other study variables. A statistically significant relationship was found between worse quality of life and illiteracy, social risk, cognitive impairment, anxiety, depression, visits to the emergency room in the previous year and medication-related factors: taking more than 10 drugs, nonadherence to treatment, medication errors, and inability to identify the medication with the disease. Conversely, a significant relationship was observed between good quality of life and a high socioeconomic level and autonomy for performing usual activities.

**Table 4:** Results on the EQ-Index (0–1) and EQ-VAS (0–100) according to the study variable.

Variable	Categories	EQ-Index^1^ (0.65±0.19)^a^	EQ-VAS^2^ (59.25±20.93)^b^

Cohabitation	Living alone	0.62±0.18	55.65±21.28
	Living with a partner	0.68±0.19^c^	60.20±21.02
	Others	0.59±0.18	60.86±20.16
Socioeconomic level	Low	0.59±0.21	47.89±22.06
	Middle	0.65±0.19	59.19±20.72^d^
	High	0.77±0.16^c^	73.24±16.01^c^
Level of education	Illiterate	0.57±0.18^c^	49.18±18.72^c^
	Literate	0.66±0.17	58.89 ^c^±20.67
	Primary studies	0.69±0.21	66.13±19.49
	High school	0.78±0.20	67.41±19.57
	University studies	0.71±0.19	79.33±14.50^e^
Social assessment (Gijón scale)	Low or normal	0.68±0.18^c^	61.34±20.40^c^
	Intermediate	0.54±0.18	48.52±20.46
BADL^3^ (Barthel test)	Independent	0.72±0.17^c^	64.65±17.84^c^
	Mild dependent	0.52±0.14	48.50±18.27
	Moderate dependent	0.49±0.11	46.84±18.76
Mental assessment (Pfeiffer test)	Normal	0.66±0.19^c^	59.84±20.71^c^
	Mild impairment	0.50±0.16	35.56±15.89
Anxiety (Goldberg scale)	Yes	0.59±0.17^c^	55.80±20.09^c^
	No	0.75±0.18	64.53±20.78
Depression (Goldberg scale)	Yes	0.56±0.18^c^	0.49±21.13^c^
	No	0.70±0.18	64.11±19.12
ER visits (previous 12 months)	No	0.70±0.18	62.63±19.67
	1–3 times	0.63±0.19^f^	57.60±21.24^f^
	>3 times	0.52±0.15^c^	46.59±22.16^c^
Number of medicines	≤10	0.71±0.18^c^	64.13±20.84^c^
	>10	0.60±0.18	53.94±19.74
Adherence (Morisky-Green)	Adherence	0.68±0.19^c^	61.44±20.99^c^
	Non-adherence	0.63±0.18	56.89±20.65
Drug-related problems (P1, P2)^4^	Yes	0.64±0.19^c^	58.04±20.92^c^
	No	0.71±0.19	65.32±19.99
C7.1- Patient forgets to use/take drug	Yes	0.63±0.18	56.45±19.86^c^
	No	0.67±0.19	61.33±21.49
C.5.5- Wrong drug taken	Yes	0.54±0.19	47.50±27.67^c^
	No	0.65±0.19	59.64±20.60
C5.6- Drug abused (unregulated overuse)	Yes	0.61±0.18^c^	56.01±21.13^c^
	No	0.69±0.19	62.53±20.24
C5.1- Inappropriate timing of administration and/or dosing intervals	Yes	0.62±0.19^c^	57.94±21.16
	No	0.69±0.19	61.42±20.41
Different brands of medicines	Yes	0.65±0.18	58.94±20.52
	No	0.63±0.22	60.93±23.05
Identifies medicines with disease	Yes	0.65±0.19	60.74±20.72^c^
	No	0.63±0.19	53.42±20.82

^1^EQ-VAS: EuroQol visual analogue scale

^2^EQ-Index: EuroQol Index

^3^BADL: Basic activities of daily living.

^4^Drug-related problems (DRP) according to the PCNE classification V6.2. P1: Treatment effectiveness (There is a (potential) problem with the (lack of) effect of the pharmacotherapy); P2: Adverse reactions (Patient suffers, or will possibly suffer, from an adverse drug event).

^a, b^(mean ± SD of EQ-I and EQ-VAS)

^c^p < 0.05 compared to other categories

^d^p < 0.05 middle socioeconomic level vs. low

^e^p < 0.05 university studies vs. other categories

^f^p < 0.05 1–3 ER visits vs. no visits.

Regarding the causes DRP, category C5.6 was the most frequently observed factor in patients with different brands of a medicine in their homes (52.2% vs. 39%, p < 0.002) and in patients who were not able to relate their medication with the disease (61.8% vs. 47.2%, p < 0.05).

Multivariate analysis **(Table 5)** showed a relationship between good quality of life and age, male sex and functional independence. Conversely, poor quality of life was associated with low educational level, social risk, taking more than 10 medications, cognitive impairment, anxiety and depression.

**Table 5:** Study variables related to quality of life (multiple linear regression).

		EQ-VAS^1^			EQ-Index^2^	
Variable	β^3^	95% CI	p	β	95% CI	p
Barthel^a^	14.27	10.19/18.35	<0.001	0.18	0.15/0.21	<0.001
Depression^b^	−10.13	−13.98/−6.29	<0.001	−0.08	−0.11/−0.05	<0.001
Social risk ^c^	−7.23	−12.17/−2.29	0.004	−0.06	−0.10/0.02	0.003
Level of education^d^	−13.58	−19.27/−7.89	< 0.001	−0.04	−0.08/−0.01	0.019
Number of medicines^e^	−4.85	−8.49/−1.20	0.009	−0.05	−0.08/−0.02	0.002
Age^f^	0.48	0.14/0.81	0.005	−0.01	−0.01/0.01	0.512
Level of education^g^	−7.40	−11.97/−2.82	0.002	0.01	−0.07/0.09	0.78
Pfeiffer^h^	−13.63	−26.06/−1.19	0.032	0.03	−0.07/0.13	0.557
Anxiety^i^	−1.27	−5.16/2.62	0.520	−0.09	−0.12/−0.06	<0.001
Sex^j^	1.01	−2.97/4.98	0.620	0.04	0.01/0.07	0.013

^1^EQ-VAS: EuroQol visual analogue scale

^2^EQ-Index: EuroQol Index

^3^Beta regression coefficient.

^a^Independent

^b^Risk of depression (Goldberg)

^c^Intermediate social risk (Gijón)

^d^Illiterate

^e^>10 medicines

^f^Older

^g^Literate

^h^Mild impairment

^i^Risk of anxiety (Goldberg)

^j^Male.

## Discussion

We investigated self-reported quality of life of a sample from a population of patients older than 65 years who used multiple medications (polypharmacy), and identified medication-related factors associated with HRQoL. We excluded people with moderate to severe cognitive impairment because their answers could not be validated through the tests. In addition, we excluded people with severe disabilities because the aim in this study was to assess HRQoL in patients who used multiple medications and were autonomous in terms of their medication management.

The average score on the EQ-VAS (59.25) in our sample matches the results of other national studies [27, 30]. Our mean score is consistent with findings from a previous study [31] of >75 year-old patients in six European countries (61.9%) which reported values ranging from 60.0 for Italy to 72.9 for The Netherlands. However, our mean EQ-VAS score is lower than that reported by Kind et al. [32] in their study of >60 year-old patients in the United Kingdom (76.9%).

There were significant sex differences in self-reported quality of life. Women generally had a worse perception of their own health status than men. This tendency was observed in the overall results for the EQ-VAS (56.43 vs. 64.19) and in all dimensions of the EQ-Index (0.62 vs. 0.72). Most of the studies we reviewed also noted differences by sex [32–34]. This suggests the possible existence of intrinsic or extrinsic factors that influence the subjective perception of loneliness and disability or affective disorders, which are prevalent problems among women in the age group we studied [13].

Our results for the influence of age were not so conclusive. Previous studies [7,13] were consistent with our finding that older people obtain higher scores on the EQ-VAS (59.47 vs. 59.07) and lower scores on the EQ-Index (0.63 vs. 0.67). We also found a lower prevalence of problems in the EQ-Index anxiety/depression dimension among older people, even although the prevalence was higher in other dimensions. This result is in agreement with other studies [31, 35], probably because elderly patients adapt to their new reality despite the appearance of new diseases and disabilities, which would explain the higher prevalence of problems in the other dimensions.

Among the EuroQol-5D dimensions, problems were seen most frequently with anxiety/depression, pain and mobility. This finding differs from those obtained by Savioa et al. [36] in a study of >75 year-old patients in Italy who reported pain, anxiety/depression and mobility as their most prevalent problems. Ruiz et al. [37] studied patients receiving antithrombotic prophylaxis and observed that the dimension most severely affected in those with severe disorder was daily routine, while the least severely affected dimension was anxiety/depression. This may be related with the initial impact of diseases on daily routines. When a disease becomes chronic, patients develop adaptation mechanisms that are steadily replaced by others as new problems arise.

The other characteristics of our study population associated with a lower quality of life were consistent with those described in the literature: low educational level [31, 38], social risk [33,37], low socioeconomic status [38], living alone [7,33], anxiety and depression [14,39], functional dependency and cognitive impairment [11, 40]. In this regard, we note that even slight differences in functional or cognitive ability significantly influenced quality of life.

In earlier research the factors that stand out, apart from patient-related factors, are those connected with medication–which may be determining factors in quality of life. In our patients the medication-related factors with a negative influence on quality of life were taking more than 10 medicines, nonadherence to treatment and the causes of DPR, mainly categories C7.1, C5.5, C5.6 and C5.1.

Most reports that relate medication to quality of life have focused on adherence. Holt et al. [19] found an association between low level of HRQoL and low adherence to antihypertensive medication among elderly patients, while Chew et al. [20] observed that better adherence had positive effects on different dimensions of the HRQoL in adults with type 2 diabetes mellitus. In addition, Saleh et al. [21], in their study of patients with type 2 diabetes, found that those with the worst levels of quality of life did not fully follow instructions for self-care and medication. One earlier study found that adherence to medication was a good predictor of better quality of life [22], and we also noted a statistically significant relationship between better adherence and a higher quality of life.

We searched for possible associations between patients’ medication errors and quality of life. Most studies that related these two variables have focused on errors made during the prescription, preparation and administration of medicines [41–43]. It is generally thought that the actors responsible for medication errors are health professionals. However, in many cases it is the patients themselves (or their caregivers) who make mistakes in the administration of medications, and these errors may cause adverse events [44].

In our study the occurrence of errors (mainly in the dose) was consistently related with a worse quality of life. We found that 83.2% of patients had some causes of DRP, with C5.1 (62.1%) and C5.6 (50.1%) being the most frequent. Previous research has reported frequencies of patients’ medication errors between 19% and 59%, with older patients making more errors than others, especially with the dosage [44].

In addition, we found that errors in taking medications, essentially dose errors, showed a statistically relevant association with the presence of different brands of medicines and the patient’s not knowing which disease they were prescribed for. Our results were consistent with those of Lisón et al. [45] in their study of elderly patients who use multiple medications.

In our study, the number of medicines was the medication-related factor that showed the strongest relation with quality of life. Patients who take a larger number of medicines reported the worst results in quality of life both in the EQ-Index and the EQ-EVA. Others have also observed a relationship between a higher number of medicines and a worse quality of life [16, 17]. Moreover, this association has been reported concomitantly with errors in taking medication and lack of adherence [10, 46]. The associations among these factors make it important to consider that patients’ problems are not always solved with new medicines. In the case of elderly patients who use multiple medications there is a danger of falling into the “prescription cascade” [47, 48], which consists of treating a previous problem caused by a prescribed medicine with a new medication.

In the final multivariate analysis, together with other patient-dependent factors such as educational level, social risk, functional capacity and emotional state, the number of medicines was the only medication-related factor that remained in the model as a determinant of quality of life. However, we note that a person’s lower quality of life is more likely to be due to a health condition they are treating with medications (and the severity of the condition) rather than to the medications themselves.

Our results suggest that steps are needed to make treatments easier for patients–in other words, to improve adherence, reduce medication errors and avoid possible effects on their quality of life. Among such measures, those found to be most effective are interventions to provide better advice to patient about their main illness, improve the relevance of treatment and adherence, and simplify dosages and scheduling in order to reduce the complexity of pharmacotherapy. Other potentially effective measures include personalized dosage systems, strategies to enhance face-to-face or phone communication between the health professional and the patient, and the use of devices to send patients reminders about their medications and doses [46,49,50].

## Strengths and limitations

One of the main limitations of this study is its cross-sectional design, which precludes the establishment of direct causal relationships. However, the identification of factors related to HRQoL, which was one of our intended goals, was well within the capabilities of this type of study.

Although some studies have reported seasonal variations in HRQoL [51], we did not consider the possible influence of local weather variations during the year. Given the mild climate throughout the year in the province of Malaga where the study was conducted, we believe that the seasonal variations were too small to have a relevant effect on our results.

Although the rate of refusal to participate was relatively high (17.5%) among the patients we invited to take part in this study, these individuals were replaced by recruiting new patients randomly from the lists initially provided by the health management units. We believe the characteristics of our participants were similar enough to rule out significant selection bias. The characteristics of patients who declined to participate were analyzed separately and found to be similar to those who agreed to participate. Moreover, the geographic distribution of those who agreed and declined to take part in the study was similar. An important consideration is that we found no significant differences in the results for any of the main variables according to place of residence, which further supports the external validity of our findings.

## Conclusions

Inherent factors within the patient such as functional incapacity, cognitive impairment and social and emotional problems were the main constraints to quality of life in our study population. Using larger numbers of medicines was associated with a worse quality of life.

We suggest that efforts to address problems in elderly patients could focus on the development of social and health strategies to enhance their maximum functional capacity and mental health, correct situations of social risk, and avoid the use of multiple medications.

## Supporting information

S1 File Appendix_Measures Instruments. (DOCX)
