# Supplementary material for: Medication-related factors associated with health-related quality of life in patients older than 65 years with polypharmacy
Source: PLoS One. 2017 Feb 6;12(2):e0171320. doi: 10.1371/journal.pone.0171320 (PMC5293190; doi:10.1371/journal.pone.0171320)
Supplement: S1 File — (DOCX) [file pone.0171320.s001.docx]

**Appendix: Measures Instruments**

**Pfeiffer**

Short Portable Mental Status Questionnaire (SPMSQ)

Instructions: Ask questions 1 to 10 on this list and record all answers. All responses must be given without reference to calendar, newspaper, birth certificate, or other aid to memory. Record the total number of errors based o the answers to the 10 questions.

Total Number of Errors

0 – 2 errors = Intact Intellectual Functioning

3 – 4 errors = Mild Intellectual Impairment

5 – 7 errors = Moderate Intellectual Impairment

8 – 10 errors = Severe Intellectual Impairment

Reference:

Pfeiffer E. A short portable mental status questionnaire for the assessment of organic brain deficit in elderly patients. J Am Geriatr Soc. 1975;23(10):433-41.

**The Barthel ADL Index: Guidelines**

1. The index should be used as a record of what a patient does, not as a record of what a patient could do.
2. The main aim is to establish degree of independence from any help, physical or verbal, however minor and for whatever reason.
3. The need for supervision renders the patient not independent.
4. A patient's performance should be established using the best available evidence. Asking the patient, friends/relatives and nurses are the usual sources, but direct observation and common sense are also important. However direct testing is not needed.
5. Usually the patient's performance over the preceding 24-48 hours is important, but occasionally longer periods will be relevant.
6. Middle categories imply that the patient supplies over 50 per cent of the effort.
7. Use of aids to be independent is allowed.

Scoring interpretation

Sum the patient's scores for each item. Total possible scores range from 0 – 100, with lower scores indicating increased disability.

0-20 Total dependence

21-60 Severe dependence

61-90 Moderate dependence

91-99 Slight dependence

100 Independence

References:

Mahoney FI, Barthel D. “Functional evaluation: the Barthel Index.” *Md Med J* 1965;14:56-61.

**Goldberg Depression and Anxiety scales**

The Goldberg Depression and Anxiety Scales give scores of 0 to 9 for the number of symptoms of depression and of anxiety. Each item is answered with a 'yes/no' response. Items are scored 0 (no) or 1 (yes) and summed.

Interpretation: Add anxiety score, add depression score. Patients with anxiety scores of five or depression scores of two have a 50% chance of having a clinically important disturbance; above these scores the probability rises sharply.

When answering the questions think back over the last seven days and try to evaluate your condition in this time span. Decide to which extent the individual statements apply to how you’re feeling.

But please note: The test result is NOT a final diagnosis.

Reference:

Goldberg D, Bridges K, Duncan-Jones P, et al. Detecting anxiety and depression in general medical settings. Br Med J 1988; 97: 897-899.

Goldberg D, Bridges K, Duncan-Jones P, et al. Detección de la ansiedad y la depresión en el marco de la medicina general. Br Med J (ed. esp.) 1989; 4(2): 49-53.

**Gijón Scale**

The degree of social support received by the patient was measured on admission according to the Gijón Socio-Family Evaluation Scale. This is an administration by proxy test that assesses socio-family risk and consists of five items (family, economic, housing and relational situation and social support) each containing five possible categories, ranging from an ideal social situation or lack of problems to the objective determination of a particular circumstance or social problem. With the global score calculated, the cut-off point for the detection of social risk is 16.

Reference:

Cabrera D, Menéndez A Fernández A, Acebal V, García JV, Díaz E, Salamea A. Evaluación de la fiabilidad y validez de una escala de valoración social en el anciano. Aten Primaria 1999; 23(7): 434-440.
